# Preparation and Optimization of Mn^2+^-Activated Na_2_ZnGeO_4_ Phosphors: Insights into Precursor Selection and Microwave-Assisted Solid-State Synthesis

**DOI:** 10.3390/nano15141117

**Published:** 2025-07-18

**Authors:** Xiaomeng Wang, Siyi Wei, Jiaping Zhang, Jiaren Du, Yukun Li, Ke Chen, Hengwei Lin

**Affiliations:** International Joint Research Center for Photo-Responsive Molecules and Materials, School of Chemical and Material Engineering, Jiangnan University, Wuxi 214122, China; 7220611009@stu.jiangnan.edu.cn (X.W.); 6240608017@stu.jiangnan.edu.cn (S.W.); 6220608024@stu.jiangnan.edu.cn (J.Z.); 6230608045@jiangnan.edu.cn (Y.L.); 6240609010@stu.jiangnan.edu.cn (K.C.)

**Keywords:** Na_2_ZnGeO_4_:Mn^2+^, microwave-assisted synthesis, green emitting phosphor

## Abstract

Mn^2+^-doped phosphors emitting green light have garnered significant interest due to their potential applications in display technologies and solid-state lighting. To facilitate the rapid synthesis of high-performance Mn^2+^-activated green phosphors, this research optimizes a microwave-assisted solid-state (MASS) method for the preparation of Na_2_ZnGeO_4_:Mn^2+^. Leveraging the unique attributes of the MASS technique, a systematic investigation into the applicability of various Mn-source precursors was conducted. Additionally, the integration of the MASS approach with traditional solid-state reaction (SSR) methods was assessed. The findings indicate that the MASS technique effectively incorporates Mn ions from diverse precursors (including higher oxidation states of manganese) into the crystal lattice, resulting in efficient green emission from Mn^2+^. Notably, the photoluminescence quantum yield (PLQY) of the sample utilizing MnCO_3_ as the manganese precursor was recorded at 2.67%, whereas the sample synthesized from MnO_2_ exhibited a remarkable PLQY of 17.69%. Moreover, the post-treatment of SSR-derived samples through the MASS process significantly enhanced the PLQY from 0.67% to 8.66%. These results underscore the promise of the MASS method as a novel and efficient synthesis strategy for the rapid and scalable production of Mn^2+^-doped green luminescent materials.

## 1. Introduction

Green emission, as one of the three primary colors in luminescent systems, plays a critical role in applications such as display technologies, solid-state lighting, and safety signage [1,2,3,4]. In recent years, a variety of high-performance green phosphors have been developed. In addition to the conventional commercial green phosphor SiAlON:Eu^2+^ [5], several novel Eu^2+^-activated green-emitting materials have been reported, such as Ba_2_LiSi_7_AlN_12_:Eu^2+^ and Ba[Li_2_(Al_2_Si_2_)N_6_]:Eu^2+^ [6,7]. Although these materials exhibit excellent optical properties, they typically require Eu^2+^ as the luminescent center and the construction of complex oxynitride lattices, necessitating harsh synthesis conditions and highly reducing environments [8,9,10,11,12]. As a result, Mn-based green phosphors have attracted increasing attention as promising alternatives. These materials utilize transition metal Mn ions as activators and offer the advantage of simpler synthesis processes [13,14].

As a multivalent, non-rare-earth ion with accessible oxidation states ranging from +2 to +7, Mn ions are of particular interest [15,16,17,18,19,20]. Green emission from Mn^2+^ is typically attributed to the spin- and parity-forbidden ^4^T_1_ → ^6^A_1_ transition of the 3d^5^ electronic configuration in specific crystal field environments [21,22,23,24]. In recent years, numerous studies have demonstrated the potential of Mn^2+^-activated phosphors for green emission. For example, D. Zhang et al. synthesized ZnAl_2_O_4_:Mn phosphors via a sol–gel method, showing efficient green emission under blue LED excitation for potential white LED applications [25]. Subsequently, Menon et al. reported green emission at 523 nm from Mn^2+^ in Zn_2_SnO_4_ prepared via a solid-state reaction (SSR) [26]. In addition, Su et al. employed a co-precipitation precursor (COP) method to prepare γ-AlON:Mn^2+^,Mg^2+^ [27] phosphors exhibiting narrow-band green emission. Although significant progress has been made in the development of Mn^2+^-based green emitting phosphors, many of the reported synthesis routes still suffer from complex processing steps and high energy consumption. In this context, it is highly desirable to explore alternative synthesis strategies that are not only simple and environmentally friendly but also capable of achieving rapid reaction rates with low energy input. The microwave-assisted solid-state synthesis (MASS) method stands out in this regard, offering a time-efficient and energy-saving approach compared to conventional high-temperature solid-state techniques, thus providing a promising route for the scalable fabrication of Mn^2+^-doped green-emitting materials.

MASS has emerged in recent years as an efficient and convenient method for the preparation of inorganic luminescent materials [28,29]. This green technique utilizes a microwave susceptor to absorb microwave energy and convert it into heat, thereby rapidly raising the temperature of the precursor. As the reaction progresses, the dielectric properties of the precursor change, enabling it to directly absorb microwave energy and accelerate the reaction process [30,31,32,33]. In previous reports, Miranda de Carvalho et al. employed the MASS method to rapidly synthesize a series of photochromic compounds, Na_8_Al_6_Si_6_O_24_(Cl, S)_2_ [34]. Subsequently, Poelman and co-workers successfully prepared a range of luminescent materials, including aluminates, germanates, and gallates, using MASS [16,35]. Since then, numerous studies have demonstrated the versatility of this method for synthesizing various luminescent phosphors. However, there are still relatively few reports on the use of MASS for the synthesis of high-efficiency Mn^2+^-doped green-emitting materials. This is mainly because MASS, as a novel synthesis method for luminescent materials, relies heavily on the intrinsic microwave absorption characteristics of the precursors. Moreover, the process optimization strategies of MASS differ significantly from those of conventional SSR methods. In the SSR approach, the sintering temperature and reaction duration can be independently adjusted to regulate the nucleation and growth of crystals. In contrast, the MASS method involves a more complex interplay of parameters, as the microwave irradiation time simultaneously influences several critical factors, including the heating rate of the microwave susceptor, the energy absorption efficiency of the precursor, and the duration of crystal growth. This interdependence increases the complexity of optimizing synthesis conditions for achieving high-performance Mn^2+^-activated phosphors using the MASS technique. Therefore, it is necessary to systematically investigate the process parameters of MASS and explore suitable precursors for the efficient synthesis of Mn^2+^-doped luminescent materials using this method. Furthermore, in order to enhance the versatility and applicability of MASS, it is of great significance to explore the integration of MASS with conventional SSR, leveraging the microwave-driven energy absorption characteristics of the materials to improve luminescence performance and broaden the scope of synthesis.

In this work, a germanate-based system—a suitable host for the MASS technique—was selected as the doping matrix. Among them, Na_2_ZnGeO_4_ was chosen due to its abundant spatial lattice sites and relatively low structural symmetry, which can provide favorable sites for Mn^2+^ ions [36]. Accordingly, a series of Mn^2+^-doped Na_2_ZnGeO_4_ green-emitting phosphors were synthesized using three different strategies: the MASS, SSR, and a combined SSR + MASS approach. The process parameters (microwave irradiation duration and dopant contents) of the MASS method were systematically optimized, and the influence of different manganese precursors on the luminescence performance was also investigated. In addition, the compatibility of MASS with different manganese precursors was investigated. The results demonstrate that MnO_2_, Mn_2_O_3_, and MnCO_3_ can all serve as effective precursors for the successful synthesis of Mn^2+^-activated green phosphors via the MASS method, yielding photoluminescence quantum yields (PLQY) of 17.69%, 7.59%, and 2.67%, respectively. Furthermore, the integration of SSR with MASS markedly improved the PLQY from 0.67% to 8.66%, confirming the capability of MASS to enhance luminescence efficiency. These results demonstrate that MASS, either independently or in combination with SSR, offers a promising pathway for the rapid and efficient synthesis of Mn^2+^-activated green-emitting phosphors, providing valuable insights for the development of high-performance luminescent materials.

## 2. Materials and Methods

### 2.1. Materials Preparation

#### 2.1.1. The MASS Samples

**Figure 1:**
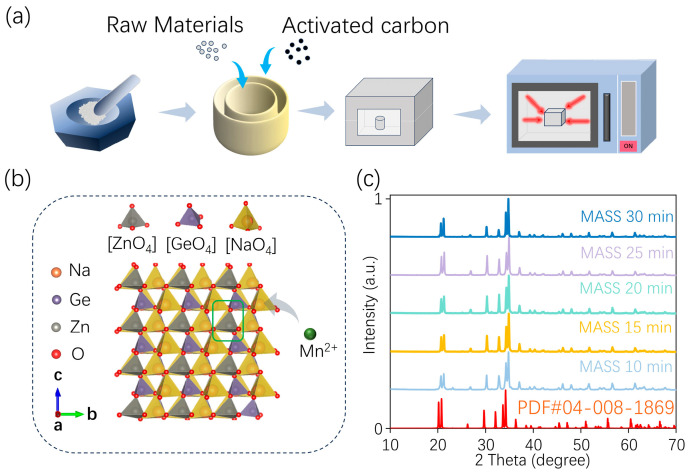
(**a**) Diagrammatic sketch of MASS method for preparing Na_2_ZnGeO_4_:Mn^2+^; (**b**) crystal structure of Na_2_ZnGeO_4_ with [ZnO_4_], [GeO_4_] and [NaO_4_] units; (**c**) XRD patterns of Na_2_ZnGeO_4_:Mn^2+^ prepared with different microwave irradiation durations (from 10 to 30 min).

As illustrated in Figure 1a, the MASS synthesis process was employed using stoichiometric amounts of ZnO (99.9%), Na_2_CO_3_ (99.99%), GeO_2_ (99.99%), and different manganese sources—MnCO_3_ (99.99%), MnO_2_ (99.99%), and Mn_2_O_3_ (99.99%)—all purchased from Aladdin without further treatment. The microwave reaction setup consisted of two alumina crucibles and aluminosilicate insulation bricks. The larger crucible (50 mL) was filled with 7 g of activated carbon (10–20 mesh, Leyan, Shanghai, China), serving as the microwave susceptor. The smaller crucible (5 mL), containing 0.8 g of precursor powder, was covered with an alumina lid and embedded into the activated carbon bed inside the larger crucible. The entire crucible assembly was placed into a cavity made from high-temperature aluminosilicate insulation bricks. The reaction was carried out in a laboratory microwave oven operating at 2.45 GHz with an output power of 700 W for 10–30 min. The resulting powders were thoroughly ground for subsequent characterization.

#### 2.1.2. The SSR Samples

Stoichiometric amounts of ZnO (99.9%, Aladdin), Na_2_CO_3_ (99.99%, Aladdin), GeO_2_ (99.99%, Aladdin), and one of the manganese sources—MnCO_3_ (99.99%, Aladdin), MnO_2_ (99.99%, Aladdin), or Mn_2_O_3_ (99.99%, Aladdin)—were carefully weighed for each synthesis batch. The resulting powder mixture was partially transferred into a 13 mm diameter die and pressed under a uniaxial pressure of 150 MPa to form the tablet precursor. These tablets, along with the remaining powder precursor, were then sintered in air at 1200 °C for 6 h in a muffle furnace, with a heating rate of 5 °C min^−1^. After sintering, the samples were furnace-cooled to room temperature, and the resulting powder was thoroughly ground into fine powders for further characterization.

#### 2.1.3. The SSR + MASS Samples

The SSR-prepared samples, synthesized using the method described previously, were subsequently used as precursors for microwave-assisted treatment following the same MASS procedure outlined earlier. In this step, 7 g of activated carbon was used as the microwave susceptor, with a microwave power of 700 W and an irradiation time of 10 min. The resulting powders were thoroughly ground to ensure uniformity and used for further characterization.

### 2.2. Structural and Morphological Characterization

The crystallographic phase purity of the obtained phosphors was examined by X-ray diffraction (XRD) using an X-ray powder diffractometer (D8, Bruker AXS GmbH, Bruker, Germany), with Cu K*α*1 (1.5406 Å) radiation (40 kV, 40 mA) at room temperature. X-ray photoelectron spectroscopy (XPS, Thermo Fisher Scientific K-Alpha, Thermo Scientific, Waltham, MA, USA) was employed to analyze the elemental composition of the samples. The microstructure and chemical composition of the samples were investigated by scanning electron microscopy (SEM; Zeiss Sigma 300, Zeiss, Oberkochen, Germany) equipped with an energy-dispersive X-ray spectroscopy system.

### 2.3. Luminescence Characterization

The room-temperature steady-state photoluminescence (PL) and photoluminescence excitation (PLE) spectra were measured by a fluorescence spectrometer (FS5, Edinburgh, UK), with a monochromated 450 W Xenon arc lamp as the excitation source. All spectra were automatically corrected for detector response. The PLQY was measured using a fluorescence spectrometer (FLS1000, Edinburg, UK) equipped with an integrating sphere coated with polytetrafluoroethylene (N-M01, Edinburgh, UK). Luminescent photos of the samples were taken with an SLR camera (T5i, Canon, Tokyo, Japan). Temperature-dependent emissions were collected from room temperature to 240 °C using a fiber connected portable spectrophotometer (Ocean Optics, Dunedin, FL, USA) and a programmed heating platform (SET450, Fanandair Co., Ltd., Shenzhen, China). The NIR spectra were measured by the NIR Quest + 1.7 900–1700 nm, equipped with the ISP-50-8-I 50MM integrating sphere (Ocean optics, Dunedin, FL, USA).

## 3. Results and Discussion

The crystal structure of Na_2_ZnGeO_4_ and the local coordination environments of Zn, Ge, and Na with four surrounding oxygen atoms are illustrated in Figure 1b. Na_2_ZnGeO_4_ belongs to the monoclinic crystal system (space group P1n1) and possesses abundant crystallographic sites. Within the crystal, three distinct coordination units are formed: Zn, Ge, and Na. Each coordinate has four oxygen atoms to form [ZnO_4_] (r_Zn_ = 0.59 Å), [GeO_4_] (r_Ge_ = 0.38 Å), and [NaO_4_] (r_Na_ = 0.59 Å), respectively. These units are interconnected via oxygen atoms to establish a three-dimensional network structure [37]. Due to the identical valence state and similar ionic radius of Mn^2+^ (r_Mn_ = 0.65 Å, CN = 4) compared to Zn^2+^, with a radius difference of less than 30%, Mn^2+^ preferentially occupies the [ZnO_4_] site to form the [MnO_4_] structure [24].

The synthesis of Na_2_ZnGeO_4_ via MASS processing differs from the conventional SSR method [24].Traditional SSR involves the direct adjustment of the heating rate, sintering temperature, and duration to regulate crystal nucleation and growth, thus facilitating the formation of pure-phase Na_2_ZnGeO_4_. In contrast, MASS relies on a reaction medium that rapidly absorbs microwave energy to heat the precursor [38,39]. As the precursor temperature increases, its ability to absorb microwave energy is further enhanced, leading to an intense reaction and rapid nucleation and growth of Na_2_ZnGeO_4_. During this process, both the reaction medium and the precursor absorb microwave energy simultaneously. However, an excessive microwave energy input may result in the overgrowth of the crystal, potentially deteriorating its performance. Therefore, it is crucial to investigate the influence of the microwave irradiation time on the structural and luminescence properties of Na_2_ZnGeO_4_:Mn^2+^. The XRD patterns of Na_2_ZnGeO_4_:Mn^2+^ synthesized using MnCO_3_ as the manganese source under varying microwave durations are presented in Figure 1c. The results show that the XRD patterns of the samples obtained under microwave irradiation for 10–30 min match well with the standard Na_2_ZnGeO_4_ phase (PDF#04-008-1869), with no detectable impurity phases. In addition, no diffraction peaks corresponding to manganese-related compounds were observed, confirming that the Na_2_ZnGeO_4_ phase can be effectively formed within this microwave duration.

**Figure 2:**
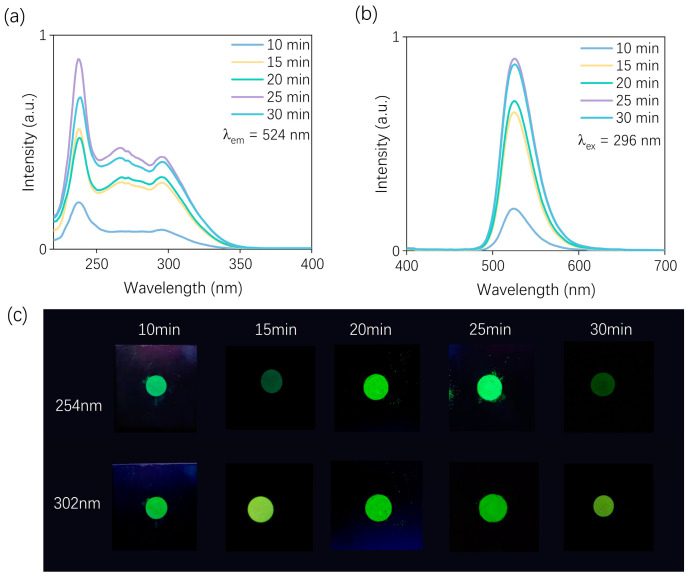
(**a**) PLE spectra of Na_2_ZnGeO_4_:Mn^2+^ samples prepared with microwave treatment (10–30 min) monitored at 524 nm; (**b**) PL spectra of the same samples under 296 nm excitation; (**c**) comparison of photographs of Na_2_ZnGeO_4_:Mn^2+^ phosphors prepared by different microwave durations upon 254 and 302 nm excitation.

Room-temperature PL and PLE spectra were further recorded to analyze in detail the effect of different microwave irradiation times on the luminescence properties of the material. As Figure 2a shows, upon monitoring at an emission wavelength of 524 nm, the excitation spectrum spans the range of 200–350 nm, featuring both a broad excitation background and several sharp peaks. The broad band in the short-wavelength region is attributed to the Mn–O charge transfer transition, while the narrow excitation peaks originate from spin- and parity-forbidden d–d transitions of Mn^2+^ ions in a tetrahedral coordination environment [40,41,42]. The PL spectra excited at 296 nm are also displayed in Figure 2b. A green emission peak centered at 524 nm with a full width at half maximum (FWHM) of 42 nm is observed, which can be attributed to the spin- and parity-forbidden ^4^T_1_ → ^6^A_1_ transition of Mn^2+^ ions. The simultaneous observation of the PLE and PL spectra reveals that both the excitation and emission intensities gradually increase with an increasing microwave irradiation time, reaching a maximum at 25 min, followed by a decline at longer durations. This observation is consistent with the previous analysis. Under short microwave irradiation durations, increasing the irradiation time facilitates improved crystal growth, thereby enhancing the luminescence performance of the material. However, prolonged microwave exposure may lead to excessive energy absorption, which can potentially alter the local crystal field environment around the luminescent centers and subsequently degrade the emission intensity. These results suggest that, within the scope of this study, there exists an optimal range of microwave irradiation time that favors enhanced luminescent performance. Excessive irradiation beyond this point may lead to deterioration in luminescent performance. Photographs of the samples under 254 nm and 302 nm UV illumination are shown in Figure 2c. The visual observations are consistent with the spectral results. Specifically, the sample irradiated for 25 min exhibits the strongest emission under UV light, displaying a vivid green luminescence. Therefore, the microwave irradiation time in this work is applied for 25 min.

**Figure 3:**
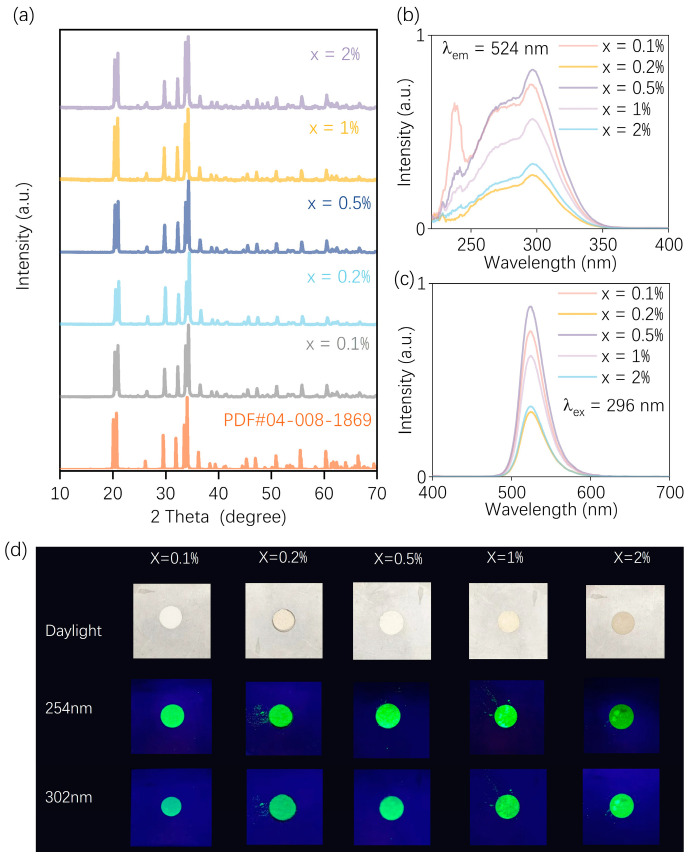
(**a**) XRD patterns of Na_2_ZnGeO_4_:Mn^2+^ prepared with different Mn^2+^ doping concentrations; (**b**) PLE spectra monitored at 524 nm and (**c**) PL spectra of the same samples excited at 296 nm. (**d**) Comparison of photographs of Na_2_ZnGeO_4_:Mn^2+^ phosphors with varying the concentrations of Mn^2+^ ions upon daylight, 254 and 302 nm excitation.

To further optimize the synthesis parameters of the MASS method, the effect of the Mn^2+^ doping concentration on the luminescence properties of the material was investigated using MnCO_3_ as the manganese source. The XRD patterns are presented in Figure 3a, and it is evident that the samples with varying doping concentrations synthesized via the microwave-assisted method exhibit a single-phase structure without detectable impurities. The elemental composition of the material was further examined through elemental mapping and XPS, as shown in Figures S1–S3. Elemental mapping (Figure S1) confirms that all target elements are successfully incorporated into the crystal structure and exhibit a uniform spatial distribution. Furthermore, the XPS spectra in Figures S2 and S3 verify that manganese ions are predominantly present in a +2 oxidation state (Mn^2+^), indicating their effective incorporation into the host lattice. This indicates that Mn^2+^ ions have been successfully incorporated into the crystal lattice across all doping levels. The PLE and PL spectra of samples with varying Mn^2+^ doping concentrations are presented in Figure 3b,c. The luminescence intensity reaches its maximum at a doping concentration of 0.5%. Notably, the MASS method differs significantly from the SSR approach. In the traditional method, when Mn^2+^ ions serve as luminescence centers, the luminescence intensity typically exhibits a systematic trend of enhancement followed by quenching as the doping concentration increases. However, in the case of MASS, the luminescence behavior appears less predictable, particularly at low doping levels, which may be attributed to local structural fluctuations induced by microwave energy absorption. Nevertheless, as the doping concentration continues to increase, concentration quenching effects begin to emerge, leading to a decline in emission intensity. Photographs of the samples with varying Mn^2+^ doping concentrations are shown in Figure 3d. All samples appear as white powders under ambient light. Under 254 nm and 302 nm UV irradiation, the samples emit bright green luminescence. Among them, the sample with a doping concentration of 0.5% exhibits the brightest luminescence, which is consistent with the photoluminescence results. Therefore, a doping concentration of 0.5%—which exhibited relatively strong luminescence performance in this study—was selected for the synthesis of subsequent samples.

**Figure 4:**
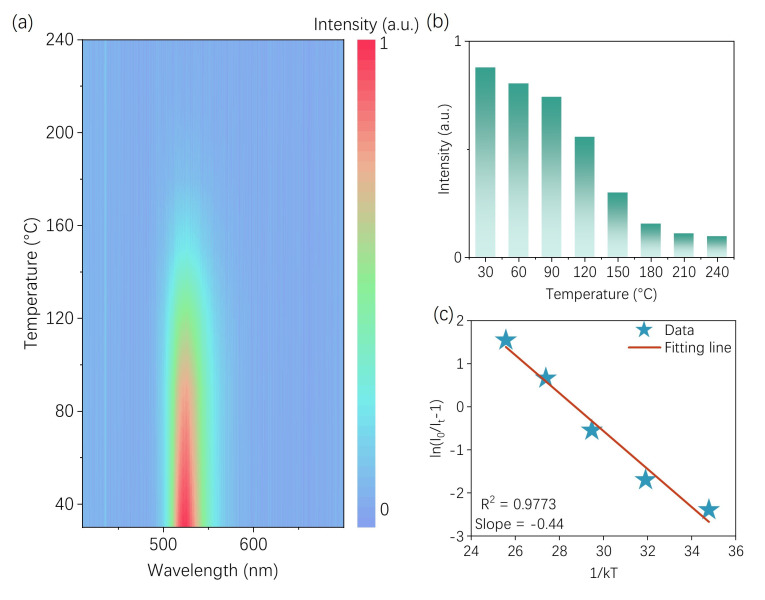
(**a**) The temperature dependent PL spectra of the Na_2_ZnGeO_4_:Mn^2+^; (**b**) the normalized integrated emission intensity of Na_2_ZnGeO_4_:Mn^2+^; (**c**) the fitting of the ln(I_0_/I_t_ − 1) as a function of 1/kT for Na_2_ZnGeO_4_:Mn^2+^.

The temperature-dependent PL spectra of the Na_2_ZnGeO_4_:Mn^2+^ upon 296 nm excitation are shown in Figure 4a. As the temperature increases, the luminescence intensity of Na_2_ZnGeO_4_:Mn^2+^ gradually decreases, as shown in Figure 4a. A quantitative analysis of the temperature-dependent emission intensity is presented in Figure 4b. It can be observed that at 150 °C, the material retains 34% of its initial emission intensity measured at 30 °C. To further investigate the underlying mechanism of thermal quenching, the thermal activation energy associated with the quenching process was calculated using the following equation [43,44]:

(1) $$I=I_{0}{\left[1+Ae^{\left(-\frac{E_{a}}{kT}\right)}\right]}^{-1},$$

where *I* represents the emission intensity at a given temperature, *I*_0_ is the initial intensity at room temperature, *k* is the Boltzmann constant (8.629 × 10^−5^ eV·K^−1^), and *T* is the absolute temperature in Kelvin. The thermal activation energy (*E_a_*) was determined from the slope of the fitted line, yielding a value of 0.44 eV. These results collectively indicate that the samples synthesized via the MASS method exhibit practically applicable thermal stability, thereby providing a foundation for the development of new materials using this approach.

Generally, since Mn^2+^ is a low-valence luminescent center, using high-valence manganese sources as precursors in air atmosphere often poses challenges. To achieve Mn^2+^ green emission during the synthesis process, high-valence Mn needs to be reduced and subsequently incorporated into the appropriate lattice sites [45,46,47,48]. For the MASS, the reaction process is more dependent on the microwave absorption characteristics of the precursors, making it more susceptible to their intrinsic properties. To evaluate the compatibility of the MASS method with different manganese sources, the luminescence properties of materials synthesized using various Mn precursors were systematically investigated. For Mn^2+^ ions, the local crystal environment has a significant influence on their emission behavior. During the MASS process, the microwave irradiation time simultaneously governs the heating of the susceptor, the energy absorption of the precursors, and the duration of crystal growth. To ensure that other synthesis parameters remained unchanged, samples were prepared using MnO_2_ and Mn_2_O_3_—serving as Mn^4+^ and Mn^3+^ sources, respectively—under the previously optimized MASS conditions and at identical doping concentrations.

**Figure 5:**
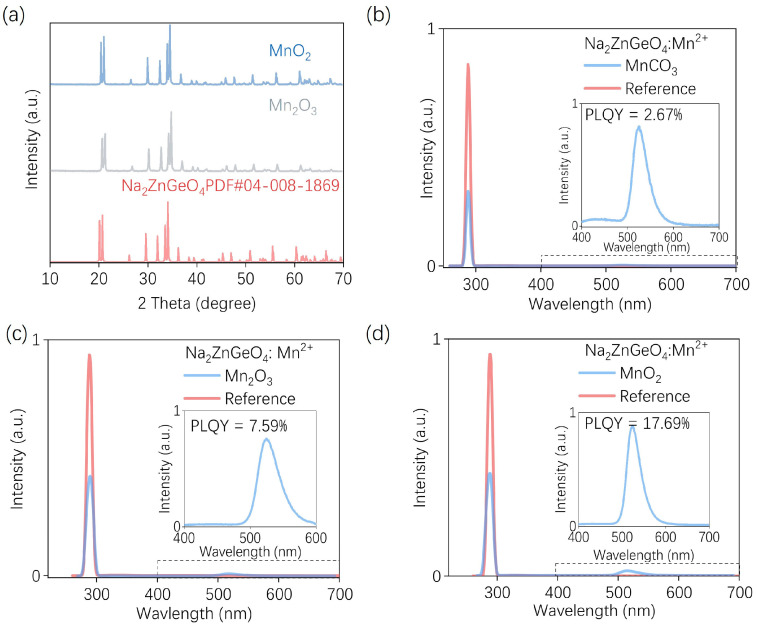
(**a**) XRD patterns of Na_2_ZnGeO_4_:Mn^2+^ prepared with precursors MnO_2_ and Mn_2_O_3_; the PLQY of Na_2_ZnGeO_4_:Mn^2+^ prepared with precursors of (**b**) MnCO_3_, (**c**) Mn_2_O_3_, and (**d**) MnO_2_.

The XRD patterns of the resulting samples are shown in Figure 5a. It is evident that all samples exhibit single-phase structures without detectable impurity peaks, indicating that the Mn ions have been successfully incorporated into the crystal lattice. PLQY measurements were further conducted to evaluate the influence of different manganese precursors on the luminescence performance of the samples. Figure 5b–d presents the emission spectra in the range of 220–700 nm, along with the corresponding magnified spectra in the 400–700 nm region. The specific quantum yield values were calculated using the following expression [49,50]:

(2) $$\eta_{QY}=\frac{\int L_{S}}{\int E_{R}-E_{S}}$$

where *L_S_* represents the integrated emission spectrum of the sample, *E_R_* is the excitation signal measured without the sample (reference), and *E_S_* denotes the excitation signal measured with the sample placed in the integrating sphere. Based on this method, the calculated PLQY values for the samples prepared by MnCO_3_, Mn_2_O_3_, and MnO_2_ are 2.67%, 7.59%, and 17.69%, respectively. It can be seen that the selected Mn compounds with +2, +3, and +4 oxidation states can all effectively provide Mn^2+^ luminescent centers during the MASS process. Although the obtained PLQY value is relatively low, the previously presented luminescence photograph under UV light demonstrates that the sample prepared with MnCO_3_ still exhibits bright green emission. Therefore, MnCO_3_, Mn_2_O_3_, and MnO_2_ can all serve as viable precursors for the preparation of Na_2_ZnGeO_4_:Mn^2+^ via the MASS method.

**Figure 6:**
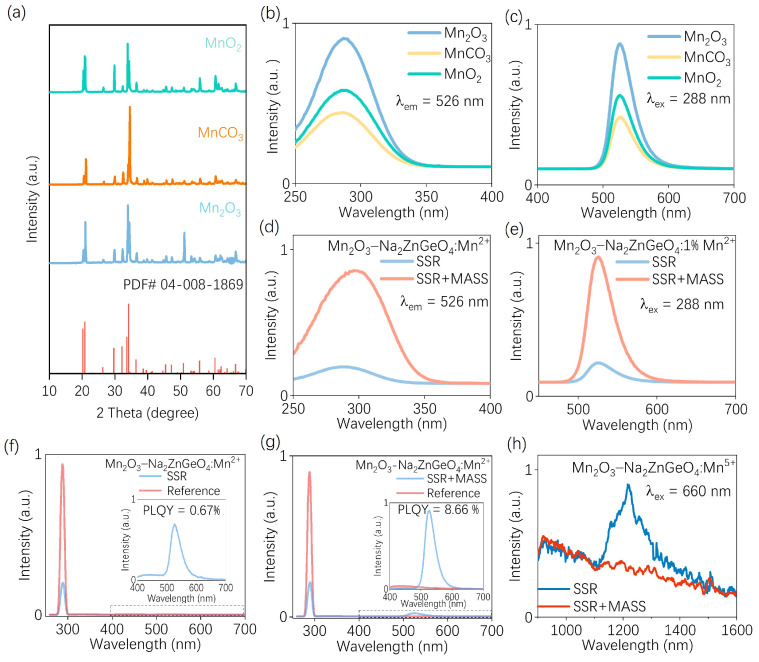
(**a**) XRD patterns of samples prepared by the SSR method using MnCO_3_, MnO_2_, and Mn_2_O_3_ as manganese sources; (**b**) PLE spectra monitored at 526 nm emission and (**c**) PL spectra excited at 288 nm of the samples; (**d**) comparison of PLE and (**e**) PL spectra of SSR samples prepared by Mn_2_O_3_ before and after a subsequent MASS processing; the PLQY of (**f**) SSR sample and (**g**) samples after subsequent MASS processing (SSR + MASS); (**h**) comparison of NIR-PL spectra of SSR sample and samples after subsequent MASS processing. The excitation wavelength at 660 nm was employed.

To further explore the combination of MASS with the conventional SSR method, a series of samples using different manganese precursors were prepared via SSR for comparison. The XRD patterns of the resulting samples are shown in Figure 6a. All samples exhibit single-phase structures, indicating that Mn ions were successfully incorporated into the crystal lattice through the SSR process as expected. The PL and PLE spectra of samples prepared using different manganese sources via the SSR method are presented in Figure 6b,c. The sample synthesized with Mn_2_O_3_ via SSR displays the highest luminescence intensity. Figure S4 shows the appearance of the sample synthesized using Mn_2_O_3_ as the precursor under sunlight and 254 nm UV irradiation. Under UV light, the sample emits a bright green luminescence, demonstrating its effective emission behavior. Therefore, the sample synthesized using Mn_2_O_3_ as the precursor was subjected to the MASS process for further investigation.

Since the MASS process relies on the absorption of microwave energy by the precursor, the reaction is initiated through bond cleavage and reorganization once the energy threshold is reached. This principle can be extended by using SSR-prepared samples as new precursors [51]. By applying a shorter microwave irradiation time, the energy input can selectively perturb the local crystal environment around Mn^2+^ without disrupting the primary phase structure, thereby modulating the luminescence properties.

The PLE and PL spectra of samples prepared using this subsequent MASS processing strategy (SSR + MASS) are shown in Figure 6d,e. A significant enhancement in emission intensity is observed after MASS treatment. To further quantify this improvement, the PLQY values of the SSR and SSR + MASS samples were measured, as shown in Figure 6f,g. The results reveal a substantial increase in quantum efficiency from 0.67% to 8.66% following the MASS post-treatment.

This enhancement is likely attributed to the ability of the MASS process to perturb high-valent Mn ions originally incorporated into the crystal lattice, thereby facilitating their redistribution into Mn^2+^-active luminescent sites and consequently improving green emission performance. To further elucidate the influence of high-valent Mn species on Mn^2+^-based luminescence, Mn^5+^ was selected for analysis due to its distinct photoluminescent behavior. Specifically, Mn^5+^ can undergo a spin-allowed ^3^A_2_ → ^3^T_1_ transition upon red-light excitation, leading to near-infrared emission around 1200 nm, which is associated with the vibrational bending mode δ(Mn–O) of the [MnO_4_]^3−^ group [52,53,54]. Moreover, previous reports have indicated a competitive relationship between Mn^2+^ and Mn^5+^ in certain host matrices [55]. As shown in Figures S5 and S6, the samples synthesized via SSR with Mn_2_O_3_ and MnO_2_ exhibit characteristic Mn^5+^ emission around 1200 nm under 660 nm excitation, confirming the formation of high-valent Mn^5+^ species. This phenomenon can be attributed to the fact that, although certain special crystal structures may facilitate the self-reduction of Mn valence states, the process is often incomplete, leading to the persistence of high-valence Mn^5+^ species, which in turn limits the optical efficiency [56,57,58,59].

Interestingly, as shown in Figure 6h, when comparing the PL spectra of SSR and SSR + MASS samples, a pronounced suppression of Mn^5+^ emission is observed following MASS treatment. This suppression may arise from two contributing factors. First, the localized structural distortion induced by microwave energy absorption during MASS may promote the occupation of Mn^2+^-favorable lattice sites. Second, the activated carbon used as a microwave susceptor generates a small amount of CO during microwave irradiation, thereby creating a mildly reducing atmosphere that facilitates the reduction of high-valent Mn^5+^ to Mn^2+^. Regardless of the exact mechanism, the net effect is a reduction in the population of Mn^5+^ occupying luminescent centers, with a corresponding increase in Mn^2+^ incorporation into optically active sites. This finding further confirms the effectiveness of the MASS technique in modulating the oxidation state and site occupancy of Mn ions to optimize the luminescence performance of phosphor materials.

**Figure 7:**
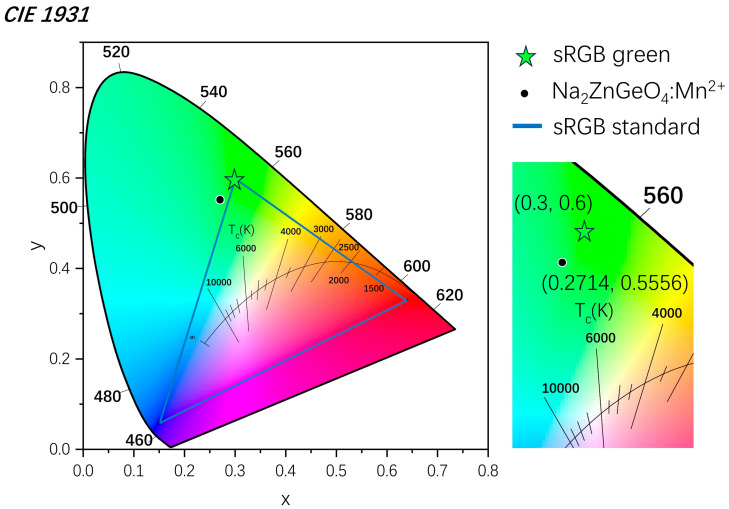
The 1931 Commission International de l’Eclairage (CIE) coordinates of Na_2_ZnGeO_4_:Mn^2+^ prepared by SSR + MASS using Mn_2_O_3_.

To further demonstrate the application potential of the SSR + MASS method in the synthesis of green-emitting phosphors, the CIE chromaticity coordinates of the sample synthesized using Mn_2_O_3_ as the manganese source were calculated, as shown in Figure 7. The coordinates were determined (0.2714, 0.5556), which are slightly shifted from the standard green coordinates of the sRGB gamut (0.3000, 0.6000), indicating a broader green emission. This deviation suggests that the emission color of the SSR + MASS-derived phosphor extends beyond the conventional sRGB green region, thereby highlighting the feasibility and potential of the SSR + MASS strategy for the development of high-performance green luminescent materials.

## 4. Conclusions

In summary, this work systematically investigated the optimization of synthesis methods and the influence of manganese precursors on the luminescence performance of Na_2_ZnGeO_4_:Mn^2+^ green-emitting phosphors. Three synthesis strategies were employed: MASS, SSR, and a combined SSR + MASS approach. The results demonstrate that MnCO_3_, Mn_2_O_3_, and MnO_2_ can all serve as effective precursors for introducing Mn^2+^ into the Na_2_ZnGeO_4_ host lattice via MASS. Among them, the sample synthesized using MnO_2_ exhibited a PLQY of 17.69%. Furthermore, combining SSR with MASS treatment significantly enhanced the PLQY of Mn_2_O_3_-derived samples from 0.67% to 8.66%. These findings provide valuable insights into the precursor adaptability of MASS and its potential for fabricating Mn^2+^-activated green phosphors with improved luminescence performance.

## Data Availability

The data supporting the findings of this study are available from the corresponding author upon reasonable request.

## Supplementary Materials

The following supplementary materials can be downloaded at: https://www.mdpi.com/article/10.3390/nano15141117/s1, Figure S1. The scanning electron microscope (SEM) image and elemental mapping of Na_2_ZnGeO_4_: Mn^2+^ prepared using MnCO_3_. Figure S2: The X-ray photoelectron spectroscopy (XPS) spectra of Na_2_ZnGeO_4_:Mn^2+^. Figure S3: High-resolution Mn XPS spectra of Na_2_ZnGeO_4_:Mn^2+^. Figure S4: The photographs of samples prepared by solid-state reaction (SSR) with Mn_2_O_3_ under daylight and 254 nm UV light. Figure S5: The photoluminescence (PL) spectra excited at 660 nm of the SSR Na_2_ZnGeO_4_: Mn^2+^ prepared with Mn_2_O_3_. Figure S6: The photoluminescence (PL) spectra excited at 660 nm of the SSR Na_2_ZnGeO_4_: Mn^2+^ prepared with MnO_2_.
