# Supplementary material for: Preparation and Optimization of Mn2+-Activated Na2ZnGeO4 Phosphors: Insights into Precursor Selection and Microwave-Assisted Solid-State Synthesis
Source: Nanomaterials (Basel). 2025 Jul 18;15(14):1117. doi: 10.3390/nano15141117 (PMC12300849; doi:10.3390/nano15141117)
Supplement: Supplementary file 1 [file nanomaterials-15-01117-s001.zip › nanomaterials-3755589-supplementary.pdf]

nanomaterials

Supporting Information

Preparation and Optimization of Mn^{2+}-Activated Na_2ZnGeO_4 Phosphors: Insights into Precursor Selection and Microwave-Assisted Solid-State Synthesis

Xiaomeng Wang †, Siyi Wei †, Jiaping Zhang, Jiaren Du *, Yukun Li, Ke Chen and Hengwei Lin *

International Joint Research Center for Photo-Responsive Molecules and Materials, School of Chemical and Material Engineering, Jiangnan University, Wuxi 214122, China; 7220611009@stu.jiangnan.edu.cn (X.W.); 6240608017@stu.jiangnan.edu.cn (S.W.); 6220608024@stu.jiangnan.edu.cn (J.Z.); 6230608045@jiangnan.edu.cn (Y.L.); 6240609010@stu.jiangnan.edu.cn (K.C.)

* Correspondence: jiaren.du@jiangnan.edu.cn (J.D.); linhengwei@jiangnan.edu.cn (H.L.)

† These authors contributed equally to this work.

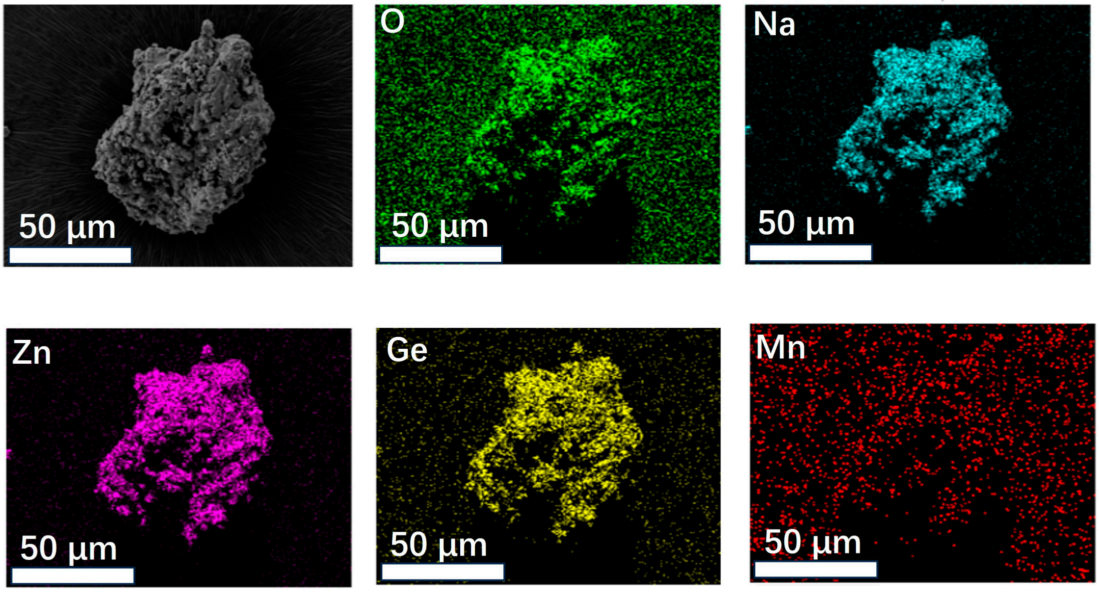

Figure S1. The scanning electron microscope (SEM) image and elemental mapping of Na_2ZnGeO_4: Mn^{2+} prepared using $MnCO_3$.

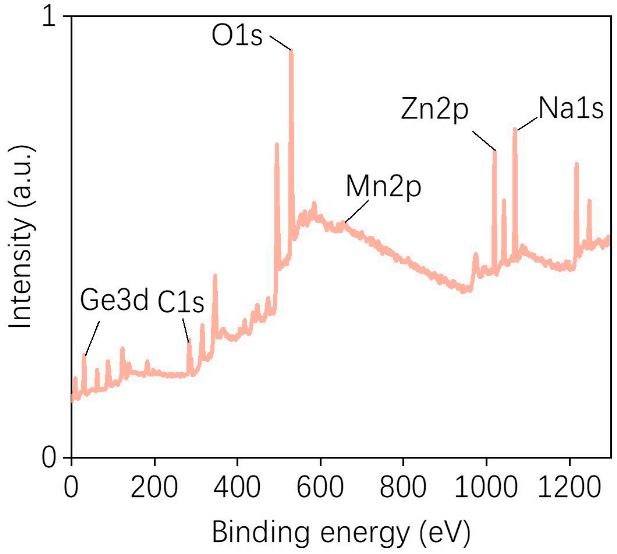

Figure S2. The X-ray photoelectron spectroscopy (XPS) spectra of $Na_2ZnGeO_4{:}Mn^{2+}$.

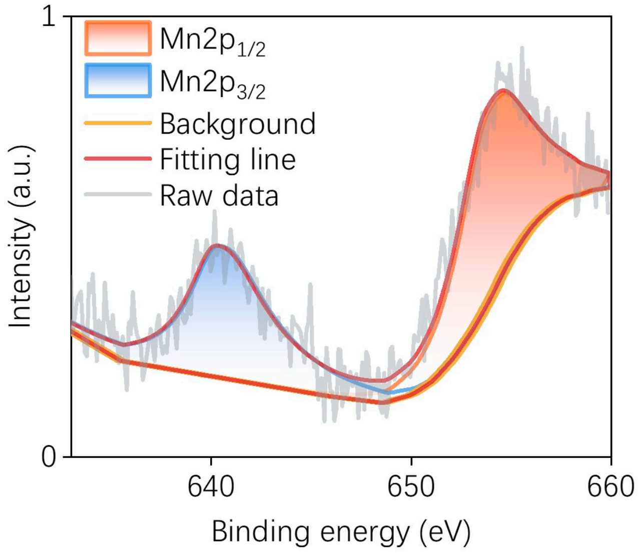

Figure S3. High-resolution Mn XPS spectra of Na_2ZnGeO_4:Mn^{2+}.

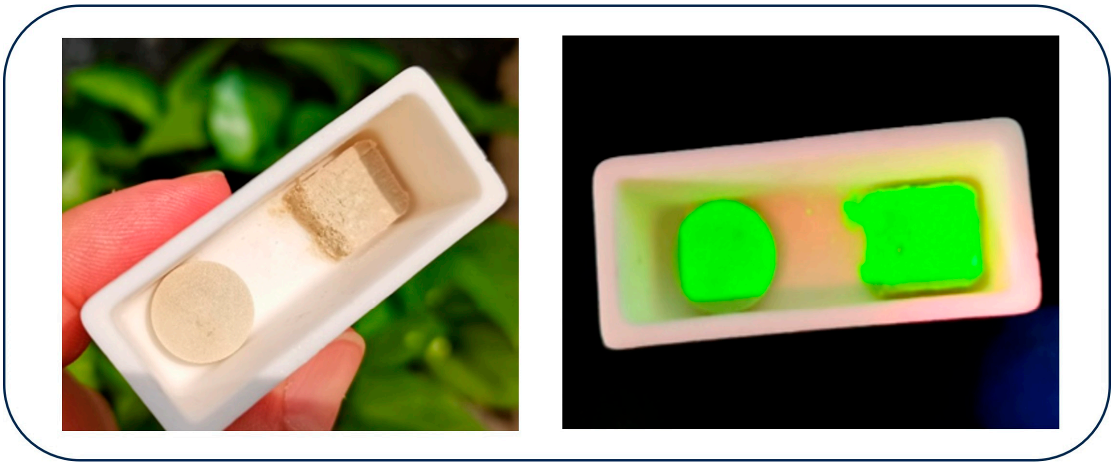

Figure S4. The photographs of samples prepared by solid-state reaction (SSR) with Mn_2O_3 under daylight and 254 nm UV light.

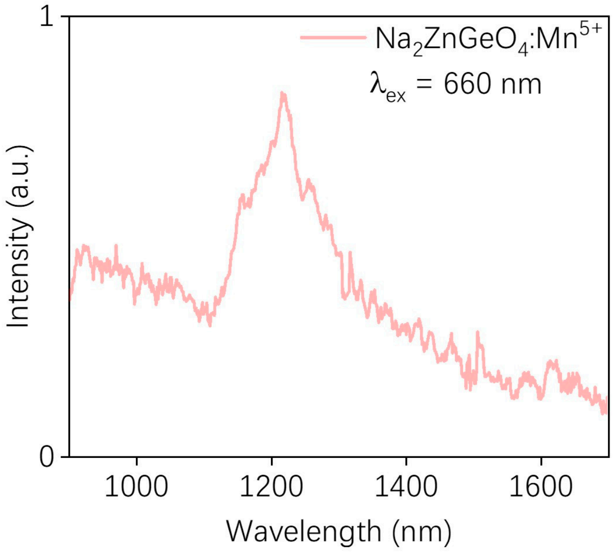

Figure S5 The photoluminescence (PL) spectra excited at 660 nm of the SSR Na_2ZnGeO_4: Mn^{2+} prepared with Mn_2O_3.

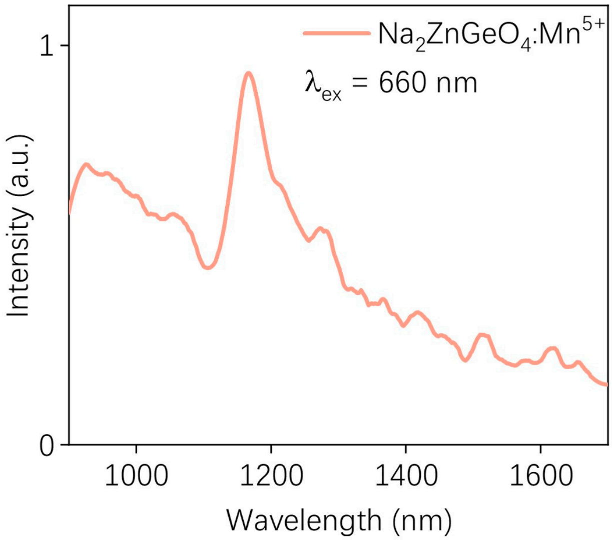

Figure S6 The PL spectra excited at 660 nm of the SSR Na_2ZnGeO_4: Mn^{2+} prepared with MnO_2.
